# Brain networks and epilepsy development in patients with Alzheimer disease

**DOI:** 10.1002/brb3.3152

**Published:** 2023-07-07

**Authors:** Dong Ah Lee, Ho‐Joon Lee, Si Eun Kim, Kang Min Park

**Affiliations:** ^1^ Department of Neurology, Haeundae Paik Hospital Inje University College of Medicine Busan Republic of Korea; ^2^ Department of Radiology, Haeundae Paik Hospital Inje University College of Medicine Busan Republic of Korea

**Keywords:** dementia, epilepsy, magnetic resonance imaging, thalamus

## Abstract

**Introduction:**

This study aimed to investigate the association between brain networks and epilepsy development in patients with Alzheimer disease (AD).

**Methods:**

We enrolled patients newly diagnosed with AD at our hospital who underwent three‐dimensional T1‐weighted magnetic resonance imaging at the time of AD diagnosis and included healthy controls. We obtained the cortical, subcortical, and thalamic nuclei structural volumes using FreeSurfer and applied graph theory to obtain the global brain network and intrinsic thalamic network based on the structural volumes using BRAPH.

**Results:**

We enrolled 25 and 56 patients with AD with and without epilepsy development, respectively. We also included 45 healthy controls. The global brain network differed between the patients with AD and healthy controls. The local efficiency (2.026 vs. 3.185, *p* = .048) and mean clustering coefficient (0.449 vs. 1.321, *p* = .024) were lower, whereas the characteristic path length (0.449 vs. 1.321, *p* = .048) was higher in patients with AD than in healthy controls. Both global and intrinsic thalamic networks were significantly different between AD patients with and without epilepsy development. In the global brain network, local efficiency (1.340 vs. 2.401, *p* = .045), mean clustering coefficient (0.314 vs. 0.491, *p* = .045), average degree (27.442 vs. 41.173, *p* = .045), and assortative coefficient (−0.041 vs. −0.011, *p* = .045) were lower, whereas the characteristic path length (2.930 vs. 2.118, *p* = .045) was higher in patients with AD with epilepsy development than in those without. In the intrinsic thalamic network, the mean clustering coefficient (0.646 vs. 0.460, *p* = .048) was higher, whereas the characteristic path length (1.645 vs. 2.232, *p* = .048) was lower in patients with AD with epilepsy development than in those without.

**Conclusion:**

We found that the global brain network differs between patients with AD and healthy controls. In addition, we demonstrated significant associations between brain networks (both global brain and intrinsic thalamic networks) and epilepsy development in patients with AD.

## Graphical abstract

Patients with AD with and without epilepsy development are similar in terms of clinical characteristics.

Global and intrinsic thalamic networks differ between patients with AD with and without epilepsy development.

Brain networks might be useful biomarkers for epilepsy development in patients with AD.

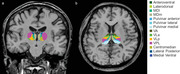

## 1 INTRODUCTION

Dementia is an acquired disorder marked by a decline in cognition in one or more cognitive domains, including learning and memory, language, executive function, complex attention, and perceptual‐motor and social cognition, which is severe enough to impede daily activities and independence (McKhann et al., 2011). Alzheimer disease (AD) is the most prevalent form of dementia in older adults, accounting for 60%–80% of cases (Caselli, 2003).

Late‐onset epilepsy, conventionally defined as the new occurrence of epilepsy in patients older than 60 years, has different clinical characteristics, including etiology, presence of epileptic auras, seizure manifestations, and features of the postictal period, than epilepsy in younger age groups (Sanya, 2010; Tanaka et al., 2013). Stroke and neurodegenerative disorders, including AD, are the main causes of late‐onset epilepsy (Sanya, 2010; Tanaka et al., 2013). Evidence suggests bidirectional associations between epilepsy and AD. Patients with AD have a higher risk of epilepsy, and late‐onset epilepsy is associated with a higher risk of AD (Dun et al., 2022). A recent meta‐analysis has shown that baseline epilepsy is associated with a higher risk of dementia (hazard ratio: 2.00) and AD (hazard ratio: 1.81). The hazard ratios for epilepsy associated with baseline dementia and AD were 2.91 and 3.11, respectively (Dun et al., 2022). Multiple studies have investigated the risk factors associated with the development of epilepsy in patients with AD and found that the duration, severity, and age at the onset of dementia may be associated with the development of epilepsy (Bernardi et al., 2010; Scarmeas et al., 2009; Sherzai et al., 2014).

Many studies have revealed that epilepsy is a brain network disease (van Diessen et al., 2014). Using brain network analysis, it is possible to localize epileptogenic lesions as well as to predict anti‐seizure medication responses and surgical outcomes in patients with epilepsy (Cho et al., 2021, 2022; Park et al., 2020). In addition, it has been found that brain network analysis can be used for the identification of patients with epilepsy or epileptogenesis (Lee, Ko, et al., 2021; Lee, Lee, et al., 2021; Li et al., 2021). One study using the intrahippocampal kainite rat model of temporal lobe epilepsy has demonstrated a significant reorganization of topographical functional brain networks in the early period of epileptogenesis, and reported that the brain network can significantly predict epilepsy development (Li et al., 2021). However, no studies have suggested that brain network changes in patients with AD are associated with epilepsy development or that epilepsy development can be predicted in advance by network analysis.

Structural covariance analysis can extend previous findings on the regional mapping of healthy and disease‐related structural brain organization by identifying complex network mechanisms. Several aspects of brain organization and development have been linked to structural covariance in both healthy subjects and patients with neurological disorders (Alexander‐Bloch et al., 2013; Larivière et al., 2022; Mechelli et al., 2005). Previous research has demonstrated a close relationship between covariance and maturational networks, indicating that these networks may reflect coordinated trophic processes throughout the brain. In addition, a close relationship between covariance network organization, heritability, and gene expression has been reported, indicating that genetic factors are likely reflected in covariance network structures (Alexander‐Bloch et al., 2013; Larivière et al., 2022; Mechelli et al., 2005). Therefore, various studies have investigated brain network abnormalities in neurological diseases using structural covariance network analysis (Larivière et al., 2022; Lee et al., 2020; Park, Lee, et al., 2018). However, there have been no studies investigating the associations between brain networks and epilepsy development in patients with AD.

This study aimed to investigate the associations between brain networks and epilepsy development in patients with AD. We hypothesized that brain network changes in patients with AD, especially changes in the global brain or intrinsic thalamic networks, explored using brain magnetic resonance imaging (MRI) and graph theory, are related to epilepsy development.

## 2 MATERIALS AND METHODS

### 2.1 Participants: patients with Alzheimer disease

**FIGURE 1:**
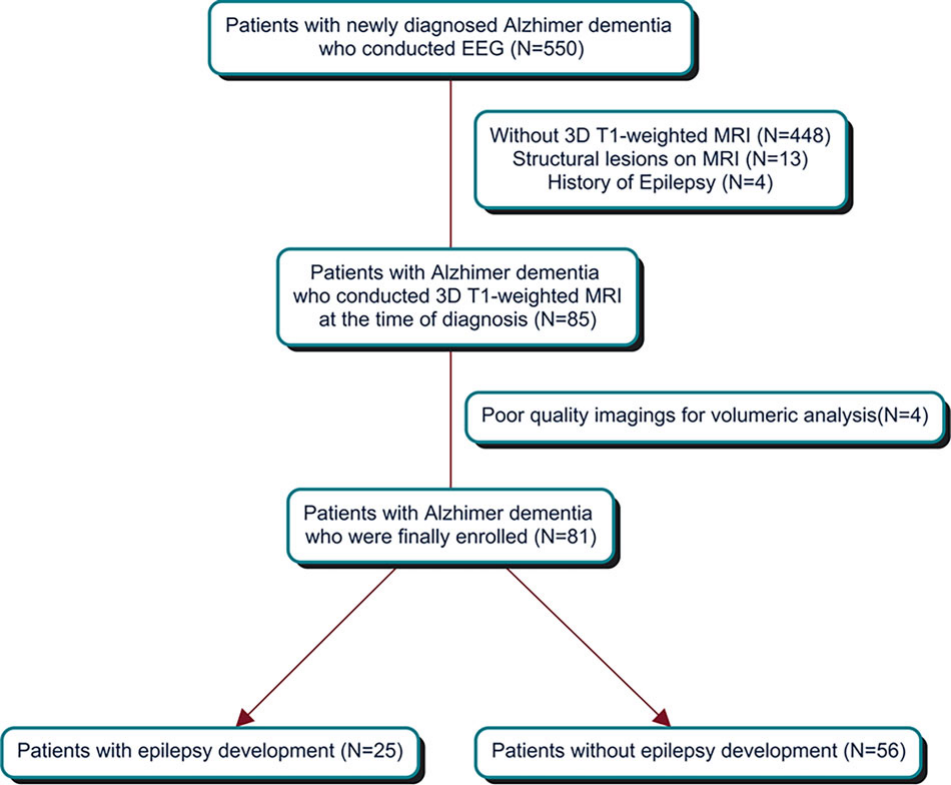
The selection process for the enrollment of patients with Alzheimer disease in this study.

This retrospective study was conducted at a single tertiary hospital and approved by the hospital's institutional review board. We enrolled patients with AD according to the following criteria: (1) they were newly diagnosed with probable AD at our hospital using the National Institute on Aging and the Alzheimer's association criteria (McKhann et al., 2011), (2) they underwent three‐dimensional T1‐weighted MRI at the time of AD diagnosis, and (3) they underwent electroencephalography (EEG) at the time of AD diagnosis. We excluded patients with (1) a history of epilepsy or epileptiform discharges on EEG, (2) structural lesions on brain MRI, or (3) poor‐quality images, which were not suitable for volumetric analysis. We divided the patients into two groups: AD patients with and without epilepsy development during the follow‐up period. Figure 1 depicts the selection process for patient enrollment in this study. We defined epilepsy development as a patient being diagnosed with epilepsy according to International League Against Epilepsy recommendation: two or more unprovoked seizures separated by at least 24 h, or at least a 60% chance of having another seizure after one unprovoked seizure (Fisher et al., 2014). The follow‐up period was the time interval between the first diagnosis of AD and the patient's last visit at our hospital.

We also enrolled healthy controls, who had no structural lesions on their MRI and no history of any medical or neurological disorders.

### 2.2 MRI acquisition

All patients with AD and healthy controls underwent MRI scanning using the same scanner (3T MRI, 32‐channel head coil, Achieva TX, Phillips Healthcare). The three‐dimensional T1‐weighted MRI had the following acquisition parameters: TI = 1300 ms, TR/TE = 8.6/3.96 ms, flip angle = 8°, and isotropic voxel size = 1 mm^3^. Fluid‐attenuated inversion recovery and T2‐weighted imaging were performed to exclude patients with structural lesions.

### 2.3 Brain network analysis using graph theory

**FIGURE 2:**
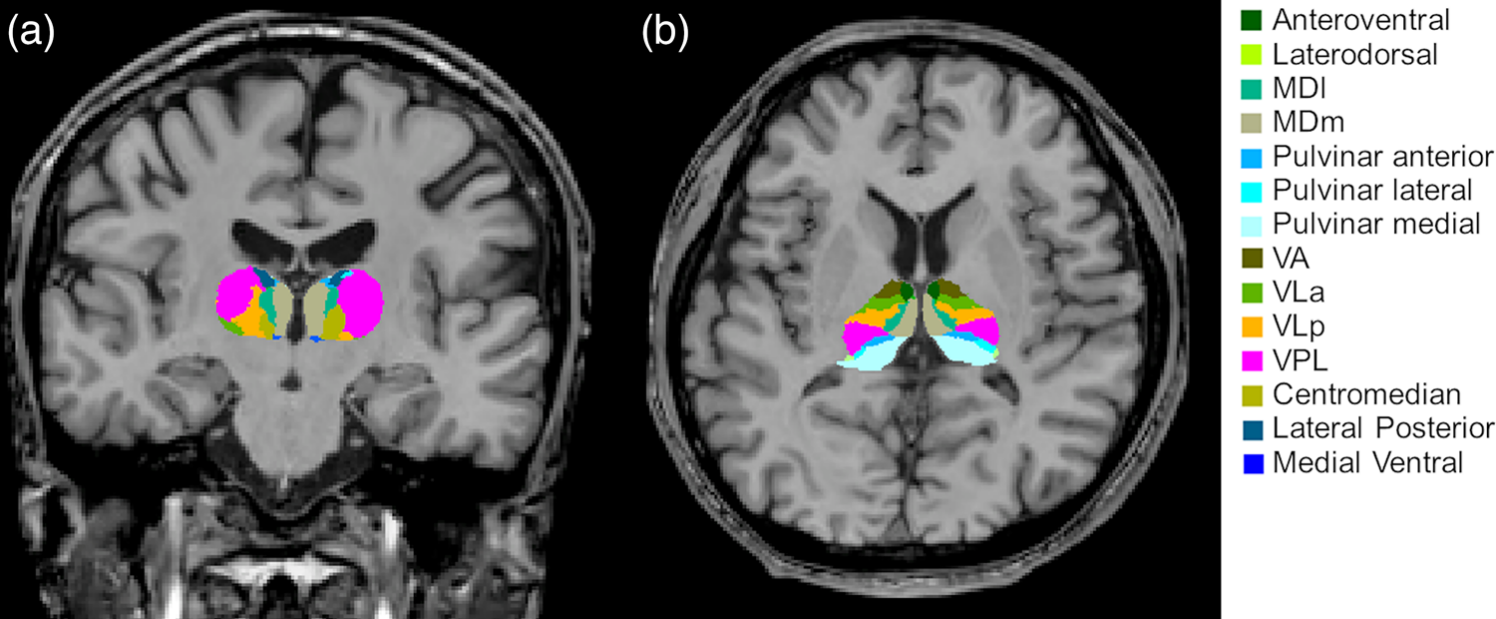
Exemplary segmentation of thalamic nuclei. Segmentations and labels of thalamic nuclei in coronal (a) and axial (b) views. Segmentations were overlaid on T1‐weighted images. MDl, mediodorsal lateral parvocellular nucleus; MDm, mediodorsal medial magnocellular nucleus; VA, ventral anterior nucleus; VLa, ventral lateral anterior nucleus; VLp, ventral lateral posterior nucleus; VPL, ventral posterolateral nucleus. *Source*: The figure was generated in courtesy of our previous study [38].

We obtained structural volumes of the cortex, subcortical structures, and thalamic nuclei using FreeSurfer. We used the “recon‐all” command to segment and obtain the structural volumes (Dale et al., 1999), and the “segmentThalamicNuclei.sh” command to segment and calculate the thalamic nuclei volumes (Supplementary 1; Figure 2), which used a combination of automated algorithms and atlas‐based priors to segment the thalamic nuclei (Greve et al., 2021). The segmentation involved identifying the boundaries between the thalamus and surrounding structures and using a series of algorithms to create probabilistic maps of the thalamic nuclei. Once the automated segmentation was completed, a series of quality control images was generated and measured to assess the accuracy of the segmentation. This allowed us to identify any errors or inaccuracies in the segmentation and make manual edits if necessary. Then, we applied graph theory to obtain the global brain network and intrinsic thalamic network based on the structural volumes using BRAPH (Mijalkov et al., 2017). Graph theory analyzed the brain networks with nodes and edges. In the global brain network, we defined the nodes as volumes in cortical and subcortical structures. In the intrinsic thalamic network, we defined the nodes as volumes in the individual thalamic nuclei. Edges were set as partial correlations among the nodes while controlling for the effects of age and sex. We set all negative correlation coefficients to zero. We created a weighted connectivity matrix using the nodes and edges and extracted network measures, such as global efficiency, local efficiency, mean clustering coefficient, characteristic path length, average strength, and assortative coefficient, from the connectivity matrix (Farahani et al., 2019; Mijalkov et al., 2017; Park et al., 2019). These measures are commonly used in brain network analysis, and they are well known to differ between healthy control groups and patients with epilepsy (Park et al., 2019; Park, Lee, et al., 2018). The global efficiency provides a measure of a graph's connectivity. A greater global efficiency indicates that information can be transmitted more quickly and easily between network nodes, indicating a greater degree of network integration. A lower global efficiency, on the other hand, suggests that the network is less connected and that information transmission may be less efficient. Local efficiency captures how interconnected a node's neighbors are. A node's high local efficiency indicates that its neighbors are well‐connected, allowing for efficient local communication even if the node is removed. A lower local efficiency, on the other hand, suggests that neighbors are less interconnected, resulting in a less efficient local communication. A greater mean clustering coefficient indicates a greater propensity for graph nodes to form clusters or tightly interconnected groups. This indicates a greater degree of local cohesion and network clustering. A lower mean clustering coefficient, on the other hand, indicates a more random or sparse connectivity pattern, with fewer clusters or local groups. A shorter characteristic path length indicates that graph nodes are more closely interconnected and that data can be transmitted more efficiently. It suggests a greater degree of global integration and efficient network communication. A longer characteristic path length, on the other hand, indicates a more dispersed or sparsely connected network in which information may take longer to reach distant nodes. A greater average strength suggests that the graph's connections have greater weights, indicating stronger relationships or interactions between nodes. The assortative coefficient quantifies the propensity of graph nodes to connect to other nodes with similar or dissimilar characteristics (Farahani et al., 2019; Mijalkov et al., 2017; Park et al., 2019). Finally, we analyzed the differences of the global brain network and intrinsic thalamic network between patients with AD and healthy controls, and we also compared the global brain network and intrinsic thalamic network between patients with AD with and without epilepsy development.

### 2.4 Statistical analysis

We compared the clinical characteristics between the groups using the Chi‐squared test, Student's *t*‐test, or Mann–Whitney test. All statistical analyses were performed using MedCalc Statistical Software version 20.111 (MedCalc Software Ltd). We used nonparametric permutation tests with 1000 permutations to determine the statistical significance of the differences between the groups in the global brain and intrinsic thalamic networks because we could obtain network measures at the group level through the BRAPH program analysis. *p*‐Values below .05 were considered statistically significant. Multiple comparison corrections were applied in the brain network analysis using the false discovery rate, Benjamini and Hochberg method (Green & Diggle, 2007).

## 3 RESULTS

### 3.1 Clinical characteristics

We enrolled 25 and 56 patients with AD with and without epilepsy development, respectively. We also included 45 healthy controls. Table 1 shows the clinical characteristics of all patients with AD and healthy controls. There were no significant group differences in age and sex between the patients with AD and healthy controls. There were no significant differences in clinical characteristics, including age, sex, education, Mini‐mental state examination (MMSE score), comorbidities, and concurrent medications between the patients with AD with epilepsy development and those without.

**TABLE 1:** Differences in clinical characteristics between patients with Alzheimer disease and healthy controls, and between patients with Alzheimer disease with and without epilepsy development.

Clinical variables	Patients with Alzheimer disease (*N* = 81)	Healthy controls (*N* = 45)	*p‐*Value
Age, years	69.9 ± 11.9	68.8 ± 10.5	.605
Male, *N* (%)	38 (46.9)	20 (44.4)	.790

Clinical variables	Patients with epilepsy development (*N* = 25)	Patients without epilepsy development (*N* = 56)	*p*‐Value
Age at diagnosis of dementia, years	67.6 ± 12.8	71.2 ± 11.6	.212
Male, *N* (%)	12 (48.0)	26 (46.4)	.897
Education, years	8.1 ± 4.4	10.3 ± 4.5	.058
MMSE score	19.2 ± 5.6	19.7 ± 6.3	.751
Comorbidities			
Vascular risk factors, *N* (%)	10 (40.4)	32 (57.1)	.156
Psychiatric disorders, *N* (%)	2 (8.0)	8 (14.3)	.43
Concurrent medications			
Acetylcholinesterase inhibitors, *N* (%)	22 (88.0)	45 (80.4)	.404
Antidepressants, *N* (%)	1 (4.0)	6 (10.7)	.324
Antipsychotics, *N* (%)	3 (12.0)	5 (8.9)	.671
Follow‐up period, months	52 (34–70)	58 (46–68)	.332
Interval between the time of dementia diagnosis and epilepsy development, months	36 (7.1–47.7)		
Seizure classification			
Generalized tonic‐clonic seizure, *N* (%)	6 (24.0)		
Focal impaired awareness seizure, *N* (%)	11 (44.0)		
Focal awareness seizure, *N* (%)	2 (8.0)		
Non‐motor seizure, *N* (%)	1 (4.0)		
Unclassified seizure, *N* (%)	5 (20.0)		

### 3.2 Brain network differences between patients with AD and healthy controls

**FIGURE 3:**
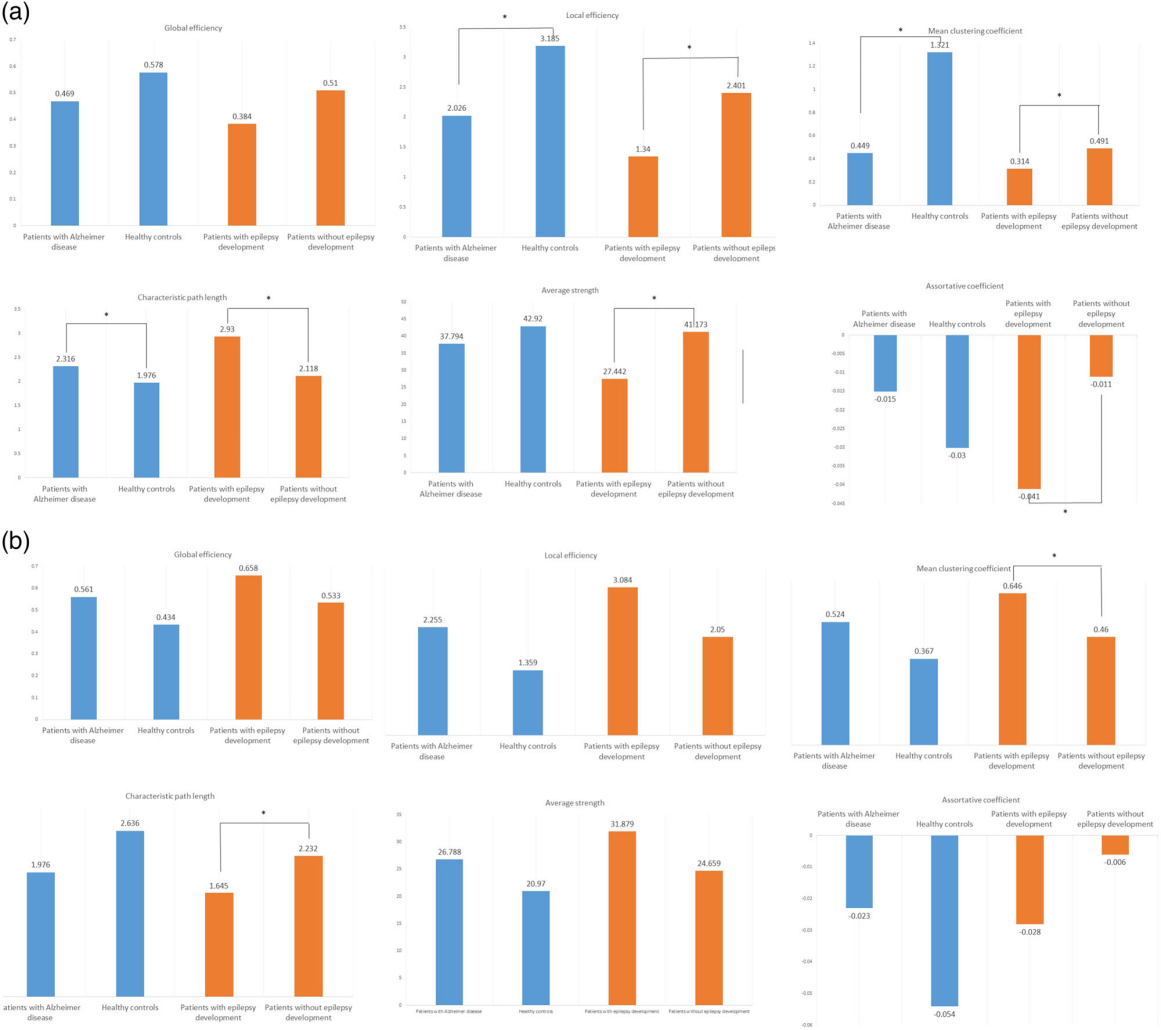
Differences in global brain network (a) and intrinsic thalamic network (b) parameters between patients with Alzheimer disease and healthy controls, and between patients with Alzheimer disease with and without epilepsy development. ^*^With statistical significance.

Tables 2 and 3 show the differences in the global brain and intrinsic thalamic networks between patients with AD and healthy controls. The global brain network was different between the groups. Its local efficiency (2.026 vs. 3.185, *p* = .048) and mean clustering coefficient (0.449 vs. 1.321, *p* = .024) were lower, whereas its characteristic path length (0.449 vs. 1.321, *p* = .048) was higher in patients with AD than in healthy controls (Figure 3). However, the intrinsic thalamic network did not differ between the groups.

**TABLE 2:** Differences in global brain network parameters between patients with Alzheimer disease and healthy controls, and between patients with Alzheimer disease with and without epilepsy development.

	Patients with Alzheimer disease (*N* = 81)	Healthy controls (*N* = 45)	Difference	CI lower	CI upper	*p*‐Value	Adjusted *p*‐value
Global efficiency	0.469	0.578	0.109	−.217	.112	.051	.306
Local efficiency	2.026	3.185	1.159	−.004	.748	.024	.048*
Mean clustering coefficient	0.449	1.321	0.872	.176	.517	.004	.024*
Characteristic path length	2.316	1.976	−0.340	−.148	3.484	.019	.048*
Average strength	37.794	42.920	5.126	1.284	3.594	.043	.051
Assortative coefficient	−0.015	−0.030	−0.150	−.048	2.564	.040	.051

	Patients with epilepsy development (*N* = 25)	Patients without epilepsy development (*N* = 56)	Difference	CI lower	CI upper	*p*‐Value	Adjusted *p*‐value
Global efficiency	0.384	0.510	0.127	−.151	.125	.048	.288
Local efficiency	1.340	2.401	1.061	−1.327	.994	.038	.045*
Mean clustering coefficient	0.314	0.491	0.177	−.150	.163	.037	.045*
Characteristic path length	2.930	2.118	−0.811	−.720	.739	.036	.045*
Average strength	27.442	41.173	13.731	−12.553	12.721	.034	.045*
Assortative coefficient	−0.041	−0.011	0.030	−.026	.025	.011	.045*

*Note*: CI: 95% confidence interval of difference between the groups.

* Statistically significant.

**TABLE 3:** Differences in intrinsic thalamic network parameters between patients with Alzheimer disease and healthy controls, and between patients with Alzheimer disease with and without epilepsy development.

	Patients with Alzheimer disease (*N* = 81)	Healthy controls (*N* = 45)	Difference	CI lower	CI upper	*p*‐Value	Adjusted *p*‐value
Assortative coefficient	−0.023	−0.054	−0.031	−.054	.014	.204	1.000
Local efficiency	2.255	1.359	−0.896	−.901	.107	.145	.174
Global efficiency	0.561	0.434	−0.127	−.144	1.140	.100	.150
Average strength	26.788	20.970	−5.818	−6.011	3.145	.087	.150
Mean clustering coefficient	0.524	0.367	−0.157	−.147	.977	.041	.123
Characteristic path length	1.976	2.636	0.660	.001	.447	.014	.084

	Patients with epilepsy development (*N* = 25)	Patients without epilepsy development (*N* = 56)	Difference	CI lower	CI upper	*p*‐Value	Adjusted *p*‐value
Assortative coefficient	−0.028	−0.006	0.023	−.043	.036	.160	.160
Local efficiency	3.084	2.050	−1.033	−1.214	.839	.085	.102
Global efficiency	0.658	0.533	−0.125	−.138	.124	.080	.102
Average strength	31.879	24.659	−7.221	−8.073	7.935	.069	.102
Mean clustering coefficient	0.646	0.460	−0.187	−.172	.159	.016	.048*
Characteristic path length	1.645	2.232	0.587	−.592	.478	.015	.048*

*Note*: CI: 95% confidence interval of difference between the groups.

* Statistically significant.

### 3.3 Brain network differences according to epilepsy development

Tables 2 and 3 present the differences in brain networks according to epilepsy development. Both global brain and intrinsic thalamic networks were significantly different between the groups. In the global brain network, the local efficiency (1.340 vs. 2.401, *p* = .045), mean clustering coefficient (0.314 vs. 0.491, *p* = .045), average degree (27.442 vs. 41.173, *p* = .045), and assortative coefficient (−0.041 vs. −0.011, *p* = .045) were lower, whereas the characteristic path length (2.930 vs. 2.118, *p* = .045) was higher in AD patients with epilepsy development than in those without (Table 2 and Figure 3). In the intrinsic thalamic network, the mean clustering coefficient (0.646 vs. 0.460, *p* = .048) was higher, whereas the characteristic path length (1.645 vs. 2.232, *p* = .048) was lower in AD patients with epilepsy development than in those without (Table 3 and Figure 3).

## 4 DISCUSSION

In this study, we found that the global brain network differed between patients with AD and healthy controls. In addition, we demonstrated significant associations between brain networks and epilepsy development in patients with AD. Both the global brain network and intrinsic thalamic network differed according to epilepsy development in patients with AD.

We found that the local efficiency and mean clustering coefficient were lower, whereas the characteristic path length was higher in patients with AD than in healthy controls, which suggests a decreased integration and segregation in patients with AD. This is consistent with a previous study (Mohammadian et al., 2022), and these results could have been expected considering the fact that cognitive function is degraded in patients with AD.

Multiple hypotheses have been proposed to explain the significant relationship between AD and the development of epilepsy. The most common origin of epilepsy in patients with AD is the temporal lobe (Tanaka et al., 2013). Seizures that occur in the temporal lobe gradually damage the hippocampus, resulting in progressive memory loss, and it has been suggested that the severity and duration of dementia may be associated with the likelihood of developing seizures (Noebels, 2011). In addition, AD is associated with an increased risk of epilepsy, especially when mutations in the amyloid precursor protein or beta‐amyloid gene pathway occur. Cabrejo et al. (2006) found 21 patients with autosomal dominant early‐onset AD who had mutations in the amyloid precursor protein locus. Dementia was observed in all cases, and seizures occurred in 12 of 21 patients (57%). It can be concluded that a genetic factor may be responsible for the overlap between dementia and epilepsy. Another possible explanation for the significant relationship between AD and epilepsy is their common molecular link. Dysregulation of kinases, such as cyclin‐dependent kinase 5, is an important pathomechanism for AD, and has also been shown in epileptogenic lesions (Sen et al., 2006). Furthermore, immune‐histochemical analyses of tissue from patients with epilepsy due to focal cortical dysplasia showed tau aggregates similar to those found in patients with AD (Sen et al., 2007).

Another recent explanation for the relationship between epilepsy and dementia is changes in brain networks. Specifically, the role of the default mode network (DMN) has attracted considerable attention. The DMN is most active at rest, whereas it is notably deactivated during goal‐directed behavior. In patients with epilepsy, DMN activation is reduced, and this reduction is correlated with the duration of epilepsy (Gonen et al., 2020). A similar pattern of decreased DMN activation has also been observed in patients with AD (Shin et al., 2011). In addition, higher scores on episodic memory and executive function tests are associated with increased functional connectivity between DMN brain regions (Dang et al., 2013).

In the present study, we focused on brain network changes in patients with AD and performed volume‐based covariance network analysis using brain MRI and graph theory. In the global brain network, the local efficiency, mean clustering coefficient, average degree, and assortative coefficient were lower, whereas the characteristic path length was higher in AD patients with epilepsy development than in those without. The local efficiency of a specific vertex is equal to the inverse of the average shortest path connecting the vertices’ neighbors (Farahani et al., 2019; Mijalkov et al., 2017; Park et al., 2019). This is related to how efficiently the brain shares information, and the higher this local efficiency value, the better is the segregation of the brain network. The mean clustering coefficient quantifies the tendency of graph nodes to cluster together, and it is positively correlated with the local efficiency (Farahani et al., 2019; Mijalkov et al., 2017; Park et al., 2019). The characteristic path length is defined as the mean number of edges in the shortest paths between every pair of vertices. This value is associated with the global efficiency of the brain, and the higher this value, the stronger is the integration of the brain network (Farahani et al., 2019; Mijalkov et al., 2017; Park et al., 2019). Therefore, our results suggest that the connectivity of the global brain network is lower in patients with AD with epilepsy development than in those without, which is consistent with the results of previous studies (Park, Lee, et al., 2018; van Diessen et al., 2014). In general, the connectivity in the global brain network is decreased in patients with focal epilepsy (Park, Lee et al., 2018; van Diessen et al., 2014).

In the intrinsic thalamic network, the mean clustering coefficient was higher, whereas the characteristic path length was lower in patients with AD with epilepsy development than in those without. We specifically focused on the thalamus in this study because it plays a pivotal role in seizure development, propagation, and termination in patients with focal epilepsy (Chaitanya et al., 2020; Cho et al., 2021; Lee et al., 2020; Park, Kim, et al., 2018; Park et al., 2019). The thalamus is a relay center for brain signaling, and the connections between different thalamic nuclei and the neocortex have been well demonstrated (Behrens et al., 2003). As a result of our intrinsic thalamic network analysis, we demonstrated that the connectivity of the intrinsic thalamic network in patients with AD with epilepsy development was significantly increased compared to that in those without epilepsy development. Our results suggest that epilepsy is a disease in which cortical excitation increases due to a low threshold for neuronal excitability, which can be induced by high synchronization of the thalamus due to increased intrinsic thalamic connectivity (Zumsteg et al., 2006).

Our study is the first to report that brain network changes are associated with epilepsy development in patients with AD, suggesting that the occurrence of epilepsy might be predictable using brain network analysis. The brain networks of patients with AD might be therapeutic targets for preventing epilepsy development. However, this study has some limitations. First, since we performed a volume‐based structural covariance network analysis instead of a structural connectivity analysis using diffusion tensor imaging, we had to obtain the brain networks at the group level instead of the individual level. Therefore, a cutoff value of the brain network changes for epilepsy development in patients with AD could not be obtained. Second, we deliberately excluded patients with previous epilepsy and epileptiform discharges on EEG at the time of AD diagnosis. Therefore, since only those who had undergone both 3D T1‐weighted MRI and EEG at the time of AD diagnosis were enrolled, this might have induced a selection bias and reduced the sample size. Third, as the thalamic nucleus is a very small structure, it is difficult to rule out the possibility that errors occurred during segmentation. However, we were able to reduce the bias that occurs during manual segmentation by using the automatic segmentation method provided by FreeSurfer. Furthermore, the automatic segmentation results showed good agreements with previous histological studies of the thalamus in terms of volumes of representative nuclei and revealed excellent test–retest reliability (Iglesias et al., 2018). Fourth, although there was no statistical difference in educational level between the AD patients with and without epilepsy development, there was a trend toward a difference. Potential influences of these education differences on the brain networks cannot be completely ruled out. Lastly, if the follow‐up period in this study was longer, the number of AD patients with epilepsy development would likely have increase, as some patients without epilepsy development would have developed it at a later timepoint.

## 5 CONCLUSION

We found that the global brain network differs between patients with AD and healthy controls. In addition, we demonstrated significant associations between brain networks (both global brain and intrinsic thalamic networks) and epilepsy development in patients with AD.

## AUTHOR CONTRIBUTIONS

*Conception and design; drafting the manuscript or revising*: Dong Ah Lee, Ho‐Joon Lee, and Kang Min Park. *Acquisition of data; analysis; and interpretation of data*: Dong Ah Lee, Ho‐Joon Lee, Si Eun Kim, and Kang Min Park. *Final approval*: Kang Min Park.

## CONFLICT OF INTEREST STATEMENT

All authors have no conflicts of interest to declare at the time of submission.

### PEER REVIEW

The peer review history for this article is available at https://publons.com/publon/10.1002/brb3.3152.

## Supporting information

Supplementary 1 The regions of nodes in the brain network.

## Data Availability

The data that support the findings of this study are available from the corresponding author upon reasonable request.
