# Supplementary material for: Brain networks and epilepsy development in patients with Alzheimer disease
Source: Brain Behav. 2023 Jul 7;13(8):e3152. doi: 10.1002/brb3.3152 (PMC10454249; doi:10.1002/brb3.3152)
Supplement: Supplementary file 1 — Supplementary 1 The regions of nodes in the brain network. [file BRB3-13-e3152-s001.docx]

**Supplementary 1.** The regions of nodes in the brain network

| **Global brain network** | |
| --- | --- |
| Brainstem |  |
| **Right hemispheric group** | **Left hemispheric group** |
| Amygdala | Amygdala |
| Caudate | Caudate |
| Hippocampus | Hippocampus |
| Pallidum | Pallidum |
| Putamen | Putamen |
| Thalamus | Thalamus |
| Accumbens | Accumbens |
| Bankssts | Bankssts |
| Caudal anterior cingulate | Caudal anterior cingulate |
| Caudal middle frontal | Caudal middle frontal |
| Cuneus | Cuneus |
| Entorhinal | Entorhinal |
| Frontalpole | Frontalpole |
| Fusiform | Fusiform |
| Inferiorparietal | Inferiorparietal |
| Inferior temporal | Inferior temporal |
| Insula | Insula |
| Isthmus cingulate | Isthmus cingulate |
| Lateral occipital | Lateral occipital |
| Lateral orbitofrontal | Lateral orbitofrontal |
| Lingual | Lingual |
| Medial orbitofrontal | Medial orbitofrontal |
| Middle temporal | Middle temporal |
| Paracentral | Paracentral |
| Parahippocampal | Parahippocampal |
| Parsorbitalis | Parsorbitalis |
| Parsopercularis | Parsopercularis |
| Parstriangularis | Parstriangularis |
| Pericalcarine | Pericalcarine |
| Postcentral | Postcentral |
| Posteriorcingulate | Posteriorcingulate |
| Precentral | Precentral |
| Precuneus | Precuneus |
| Rostral anterior cingulate | Rostral anterior cingulate |
| Rostral middle frontal | Rostral middle frontal |
| Superior frontal | Superior frontal |
| Superior parietal | Superior parietal |
| Superior temporal | Superior temporal |
| Supramarginal | Supramarginal |
| Temporal pole | Temporal pole |
| Transverse temporal | Transverse temporal |
| **Intrinsic thalamic network** | |
| **Left thalamic group** | **Right thalamic group** |
| Anteroventral | Anteroventral |
| Laterodorsal | Laterodorsal |
| Lateral posterior | Lateral posterior |
| Ventral anterior | Ventral anterior |
| Ventral anterior magnocellular | Ventral anterior magnocellular |
| Ventral lateral anterior | Ventral lateral anterior |
| Ventral lateral posterior | Ventral lateral posterior |
| Ventral posterolateral | Ventral posterolateral |
| Ventromedial | Ventromedial |
| Central medial | Central medial |
| Central lateral | Central lateral |
| Paracentral | Paracentral |
| Centromedian | Centromedian |
| Parafascicular | Parafascicular |
| Paratenial | Paratenial |
| Medial ventral | Medial ventral |
| Mediodorsal medial magnocellular | Mediodorsal medial magnocellular |
| Mediodorsal lateral parvocellular | Mediodorsal lateral parvocellular |
| Lateral geniculate | Lateral geniculate |
| Medial geniculate | Medial geniculate |
| Suprageniculate | Suprageniculate |
| Pulvinar anterior | Pulvinar anterior |
| Pulvinar medial | Pulvinar medial |
| Pulvinar lateral | Pulvinar lateral |
| Pulvinar inferior | Pulvinar inferior |
